# Dual inhibition of BDNF/TrkB and autophagy: a promising therapeutic approach for colorectal cancer

**DOI:** 10.1111/jcmm.13181

**Published:** 2017-06-09

**Authors:** Clément Mazouffre, Sophie Geyl, Aurélie Perraud, Sabrina Blondy, Marie‐Odile Jauberteau, Muriel Mathonnet, Mireille Verdier

**Affiliations:** ^1^ Laboratoire EA 3842 Homéostasie cellulaire et Pathologies Faculté de Médecine et de Pharmacie Université de Limoges Limoges Cedex France; ^2^ CHU de Limoges Service de chirurgie digestive générale et endocrinienne Limoges Cedex France

**Keywords:** colorectal cancer, neurotrophins, autophagy, therapy

## Abstract

Colorectal cancer (CRC) is the most common digestive cancer in the Western world. Despite effective therapies, resistance and/or recurrence frequently occur. The present study investigated the impact of two survival pathways—neurotrophic factors (TrkB/BDNF) and autophagy—on cell fate and tumour evolution. *In vitro* studies were performed on two CRC cell lines, SW480 (primary tumour) and SW620 (lymph node invasion), which were also used for subcutaneous xenografts on a nude mouse model. In addition, the presence of neurotrophic factors (NTs) and autophagy markers were assessed in tissue samples representative of different stages. On the basis of our previous study (which demonstrated that TrkB overexpression is associated with prosurvival signaling in CRC cells), we pharmacologically inhibited NTs pathways with K252a. As expected, an inactivation of the PI3K/AKT pathway was observed and CRC cells initiated autophagy. Conversely, blocking the autophagic flux with chloroquine or with ATG5‐siRNA overactivated TrkB/BDNF signaling. *In vitro*, dual inhibition improved the effectiveness of single treatment by significantly reducing metabolic activity and enhancing apoptotic cell death. These findings were accentuated *in vivo,* in which dual inhibition induced a spectacular reduction in tumour volume following long‐term treatment (21 days for K252a and 12 days for CQ). Finally, significant amounts of phospho‐TrkB and LC3II were found in the patients’ tissues, highlighting their relevance in CRC tumour biology. Taken together, our results show that targeting NTs and autophagy pathways potentially constitutes a new therapeutic approach for CRC.

## Introduction

CRC represents the second cause of cancer‐related mortality in developed countries 1. In accordance with the UICC TNM classification 2, CRC is categorized into five stages (0–IV), which expand into several subdivisions according to the degree of severity and metastasis. Whereas surgery and chemotherapy are more efficient on low‐grade stages, there is no curative treatment for patients with advanced‐stage CRC, and mortality remains very high because of the resistance of cancer cells, resulting in treatment avoidance 3. This aggressiveness could rely on uncontrolled growth factors pathway activation. Among them, neurotrophins (NTs), that is NGF, BDNF, NT3, NT 4/5, initially characterized in the nervous system 4, bind a specific high‐affinity, receptor—called Trk (tropomyosin receptor kinase) type A, B and C respectively. Each subtype occurs as full‐length (145 kD) or truncated (95 kD) forms. NTs primarily promote cell survival and differentiation 5 by activating either the PI3K/AKT (phosphatidylinositide 3 kinase/Akt) and/or MAPK (mitogenic activated protein kinase) and/or the PLC‐γ (phospholipase C gamma) pathway 6. TrkB expression and activation have been described for several types of cancers such as lymphomas, breast, lung cancers as reported by our team and others 7, 8, 9, 10, 11, 12, 13, 14. For CRC, we previously identified the BDNF/TrkB axis as a prosurvival pathway 7, as already been reported by Brunetto de Farias *et al*. 15. Given its description as a potential target for anticancer therapy 16, 17, the use of the Trk inhibitor K252a 18 might result in the inactivation of Trk signaling and induce a cellular response including cell death 10, 19. However, its effectiveness could be reduced by other cell survival mechanisms. Among these mechanisms, autophagy should be considered because it shares a common actor with TrkB signaling, the mTOR protein, downstream of the PI3K/AKT pathway.

Autophagy is a self‐recycling process occurring in eukaryotic cells to maintain homeostasis under basal conditions which is greatly enhanced under stress 20. It allows the degradation of altered cellular components *via* sequestration in autophagosomes, which merge with lysosomes to form the autophagolysosome 21, 22. Several specific autophagic genes called *ATG* (AuTophagy‐related Genes) are involved in the active process. The most important are *ATG6* (called Beclin1 in mammals), *ATG5* and *ATG8*, called microtubule‐associated protein light chain 3 or LC3 in mammals 23. When LC3‐I (cytosolic) combines with the autophagosome membrane, it is converted into the phosphatidylethanolamine‐linked (PE) autophagosomal form — LC3II —, which is a commonly used autophagic marker 24, 25. Although the precise role of autophagy in cancer remains ambiguous, it is considered as a prosurvival process, allowing cell retention under stress conditions (metabolic or oxidative) especially in late stages. 26. Furthermore, autophagy could be responsible for the resistance of cancer cells to therapy, as already reported for CRC 26, 27, 28. Therefore, its inhibition could assist in promoting cancer cell death 26, 27, 28 and represents a major challenge for the improvement of existing treatments 29, 30. Indeed, a recent study showed that inhibition of autophagy prevents the development and progression of CRC in patients 31.

The present study investigated the dual role of NTs and autophagy pathways in two CRC cell lines representative of different stages. The therapeutic potential of twin inhibition evidenced *in vitro* was checked and magnified *in vivo* on grafted animal. On the basis of our preliminary results, which confirmed the activation of both pathways in patients, this double therapeutic inhibition could be of considerable interest in the CRC treatment.

## Materials and methods

### Reagents and antibodies

K252a (Alomone Labs, Jerusalem, Israel), chloroquine (CQ) and rapamycin (Sigma‐Aldrich, St. Quentin Fallavier, France) were used for NT, autophagy and mTOR inhibition, respectively. The primary antibodies used for Pan‐AKT, phospho‐AKT, mTOR, phospho‐mTOR, Beclin‐1, ATG5, LC3 and cleaved caspase‐3 were obtained from Cell Signaling Technology (Saint Quentin Yvelines, France). Other primary antibodies were TrkB (BD Biosciences, Paris, France), phospho‐TrkB (Millipore, Fontenay‐Sous‐Bois, France), BDNF (Santa Cruz Biotechnology, Heidelberg, Germany), PARP (Santa Cruz), VEGF (Abcam, Paris, France), Ki67 (Ventana‐Roche, Meylan, France) and actin (Sigma‐Aldrich).

Secondary antibodies were goat HRP conjugated with anti‐rabbit IgG (Dako, Les Ulis, France) or with antimouse IgG (Dako). Others were dyed with Alexa Fluor 594 nm conjugated with anti‐rabbit IgG (Thermofisher Scientific, Waltham, MA USA) or with Alexa Fluor 488 nm conjugated with antimouse IgG. DAPI was purchased from Sigma‐Aldrich.

### Cell culture, transfection and treatments

SW480 and SW620 were obtained from American Type Cell Collection (ATCC/LGC promochem, Molsheim, France), authenticated by the seller and cultured as previously described 7 in RPMI 1640 medium (Gibco Life Technologies, Paisley, UK). SW480 was a primary CRC‐derived cell line (*i.e*. stage II), whereas SW620 was derived from the lymph node (*i.e*. stage III) of the same patient. Cells were used when they reached 80% subconfluence. Cell cultures were used for a maximum of 10 passages.

INTERFERIN transfection reagent (Polyplus; Cell Signaling Technology) was used for siRNA transfection. In total, 200,000 cells per well were transfected in 6‐well plates with either 20 nM of negative control or ATG5 siRNA (Cell Signaling Technology). After 72 hrs with fresh medium, the cells were washed and recovered as described before. Silencing was confirmed by Western blotting.

### Metabolic activity analysis

Metabolic activity was determined with the MTT assay (CellTiter 96 Aqueous One solution Cell Proliferation Assay, Promega, Charbonnières‐les‐Bains, France) following supplier instructions.

### Apoptosis and necrosis analysis

The propridium iodide (PI) / Annexin‐V‐FITC double staining method was used as previously described 10 to determine early and late apoptosis as well as necrosis rates. Analyses were performed with a FACS Calibur flow cytometer (Becton Dickinson, Heidelberg, Germany).

### Western blot analysis

After appropriate treatments, cells were collected, incubated for 15 min. at 4°C with RIPA lysis buffer, sonicated and centrifuged for 20 min. at 14,000×*g* at 4°C. Alternatively, 30 mg of tumoural tissue from patients or from engrafted mice were homogenized with ceramic bead in RIPA buffer, using Precellys mixer (MK 14 Precellys, Bertin Technology, Toulouse, France; 5500 rotations/sec.) and then centrifuged. Supernatants were collected, and the protein concentration was determined with the Bradford assay (Sigma‐Aldrich). Western blot analysis was conducted as described before 7. Band intensities were determined by densitometry using ImageJ software (NIH, Bethesda, MD, USA).

### Indirect immunofluorescence

The experiments were realized as described previously 7 and analysed using confocal microscopy (LSM 510 META; Zeiss, Göttingen, Germany). Images were processed with the ZEN software application, and surface plots of the fluorescence data were generated with the image processing program ImageJ.

### Quantitative RT‐PCR

RNA extraction was performed with the RNeasy mini kit according to manufacturer recommendations (Qiagen, Courtaboeuf, France). A high‐capacity cDNA reverse transcription kit (Thermofisher) was used and PCR reactions were realized with TaqMan Fast Universal PCR Master Mix (Thermofisher) with appropriate primers and probes as described previously 7. Results were obtained with the StepOnePlus Real Time PCR System (Thermofisher). All cycle thresholds (Ct) were normalized to *HPRT1* transcripts, and the relative quantities were calculated (untreated cells *versus* treated cells).

### Mice xenografts and treatments

All animal studies were conducted in accordance with the guidelines established by the regional Institutional Animal Care and Use Committee (Comité Régional d'Ethique sur l'Expérimentation Animale du Limousin n°87‐005). Seven‐week‐old female NMRI‐nude mice (Janvier Labs, St Berthevin, France) were subcutaneously injected with 1 × 10^6^ cells. Weekly length (L) and width (W) measurements were taken to track tumour growth, and the tumour volume was determined with the following formula: volume = [L × W(L + W)]π/12.

Once the tumour volume reached 300 mm^3^ (day 0), mice were divided into groups (control, K252a, CQ, and K252a+CQ), with *n* > 3 mice per group. A physiological saline solution of K252a (0.5 mg/kg) was administered intraperitoneally every 3 days for 21 days as described in 32. CQ was administered intraperitoneally (10 mg/kg) every 2 days for 12 days, following a modified version of the protocol used in 33. Non‐treated mice received a PBS injection every 3 days. At the end of the treatment, the animals were killed and the tumours were divided one part for Western blot analysis and the other for histological analysis.

### Histological analysis

Engrafted mice tumours were fixed in 10% formol before being dehydrated and included in paraffin following an automatic cycle (Anatomopathology department from the Limoges’ Hospital). Four micrometre paraffin sections were realized and stained with haematoxylin‐eosin‐safran (HES), to evaluate necrotic areas. For proliferation rate assessement, Ki67 staining was performed as previously described 34 using BenchMark Technology (Ventana Medical Systems; Anatomopathology department from the Limoges’ Hospital). Images were acquired with the Nanozoomer Digital Pathology 2.ORS software.

### Patient samples

A total of 12 patients (men from 70 to 88 years old), who underwent CRC resection at Limoges University Hospital (France) between March 2011 and April 2014, were enrolled in this retrospective study, according to the Declaration of Helsinki. Exclusion criteria were applied as described before 35. Tissue collection was approved by the local ethics committee (‘Comité de Protection des Personnes, Sud Ouest Outre Mer’; CPP SOOM4; number DC‐2008‐604).

### Statistics

All analyses were performed with Statview 5.0 software (Abacus Concepts, Piscataway, NJ, USA) and the anova test. A *P*‐value of <0.05 was considered statistically significant.

## Results

### Inhibition of BDNF/TrkB signaling induced autophagy in CRC cell lines

**Figure 1:**
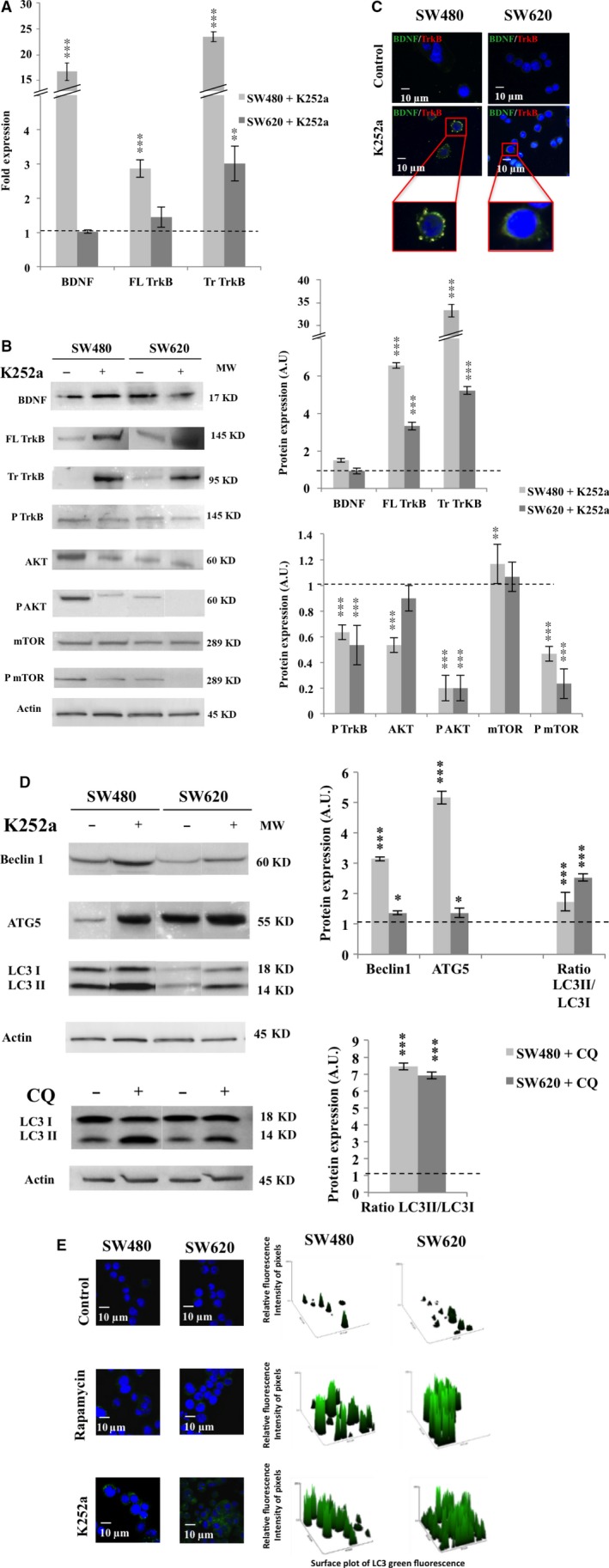
Effect of tyrosine kinase receptor inhibitor (K252a) on TrkB/BDNF expression and autophagy induction. SW480 and SW620 were treated with 100 nM of K252a for 3 hrs, recovered, washed and total RNA and proteins were extracted (see Materials and methods). (**A**) BDNF, Full Length (FL) and Truncated (Tr) TrkB, transcripts expression was assessed by qPCR. Histograms are representative of three independent experiments. The fold expression was obtained by the comparative cycle threshold method using the GAPDH RNA expression level as internal control. (**B**) BDNF, FL TrkB, Tr TrkB, phospho‐TrkB (P TrkB), AKT, phospho‐AKT (P AKT), mTOR and phospho‐mTOR (P mTOR), proteins expression were assessed by Western blotting. The density of each band representative for protein expression was calculated with ImageJ software. Images show representative results of three experiments. Statistically results are explained in comparison with control cells, normalized at 1 (*P < 0.05; **P < 0.01; ***P < 0.001). (**C**) Staining of SW480 and SW620 was realized with anti‐BDNF antibody (green), anti‐TrkB antibody (red) and DAPI (blue). Images were obtained through confocal microscopy (magnification ×1000). (**D**) Beclin1, ATG5 and LC3 proteins expression was assessed by Western blotting after treatment with either K252a (100 nM, 3 hrs) or with CQ (25 μM, 3 hrs). The density of each band representative for protein expression was calculated with ImageJ software. Images show representative results of three experiments. Statistically results are explained in comparison with control cells, normalized at 1 (*P < 0.05; **P < 0.01; ***P < 0.001). (**E**) Staining of SW480 and SW620 was realized with anti‐LC3 antibody (green) and DAPI (blue) through indirect immunofluorescence. Rapamycin (20 nM, 3 hrs) was used as a positive control for autophagy induction. Relative fluorescence quantification was accomplished by using a green surface plot with the ImageJ software application.

In a previous study, we demonstrated that CRC cells activate a survival and proliferative loop through BDNF/TrkB signaling 7; here we investigated the consequences of NT inhibition in SW480 and SW620 cells cultured for 3 hrs with 100 nM K252a. As in these cell lines, we failed to detect TrkA and TrkC expression (neither transcripts nor proteins, data not shown and 7), this pharmacological inhibitor mainly targeted TrkB signaling. Compared with control cells, a significant increase in the expression of BDNF transcripts (×16.8, *P* < 0.001), TrkB transcripts encoding the full length (145 kD, ×2.9, *P* < 0.001) and the truncated form (95 kD, ×24, *P* < 0.001) were observed in SW480 (*i.e*. CRC stage II) treated with K252a (Fig. 1A). For SW620 (*i.e*. CRC stage III), a similar significant enhancement was only observed for the truncated TrkB (×3, *P* < 0.01). Protein levels confirmed the qPCR results for both TrkB forms, in both cell lines (full length, ×6 in SW480 and ×3 in SW620, *P* < 0.001; truncated, ×35 for SW480 and ×5 for SW620, *P* < 0.001; Fig. 1B). The elevated transcription rate for BDNF observed for SW480 cell line was slightly confirmed by Western blot analysis (Fig. 1B). As both technics evaluated intracellular BDNF, we assessed the secreted form and its ability to bind TrkB. BDNF quantification in the culture medium failed to exhibit significant variations between treated and control conditions in both cell lines (data not shown). However, colocalization of BDNF with TrkB was increased after K252a treatment, especially in SW480 cells (Fig. 1C), suggesting that BDNF was able to interact with TrkB, even in the presence of K252a. Nevertheless, this enhanced receptor expression as well as the BDNF/TrkB membrane colocalization failed to activate the downstairs pathway, as evidenced by the decrease in phospho‐TrkB, phospho‐AKT and phospho‐mTOR (*P* < 0.001 for both cell lines and for the three proteins, Fig. 1B), highlighting the inhibition of this pathway, which we expected to occur with K252a.

As we previously reported for glioblastoma (Jawhari *et al*., 2016; manuscript in revision), autophagy can substitute to NTs signaling in a short time (<24 hrs). Here, a decrease in mTOR phosphorylation during K252a treatment (Fig. 1B) was compatible with autophagy activation. This result was accompanied by an increase in Beclin1 (×3, *P* < 0.001 for SW480; ×1.3 *P* < 0.05 for SW620) and ATG5 expression (×5, *P* < 0.001 for SW480; ×1.2, *P* < 0.05 for SW620; Fig. 1D). In addition, we demonstrated the conversion of cytosolic LC3 I into PE‐linked LC3 II, one of the most widely used autophagic markers (LC3‐II/LC3‐I, ×2 for both cell lines, *P* < 0.001; Fig. 1D). These results were confirmed through punctate localization of endogenous LC3 to autophagosomes with K252a treatment (Fig. 1E). By using an inhibitor of autophagic flow, CQ, we evidenced that autophagy was a functional process, as confirmed by an increased LC3II/LC3I ratio (*P* < 0.001 for both cell lines; Fig. 1D). In conclusion, during TrkB/BDNF inhibition, a functional autophagic process was activated. SW480 cells seemed to be more likely to undergo autophagy than SW620 cells. Because the inhibition of NTs signaling potentiated autophagy, we investigated whether the reverse was true and we explored the TrkB/BDNF activation when autophagy was inhibited.

### Inhibition of autophagy induced BDNF/TrkB pathway activation in CRC cell lines

**Figure 2:**
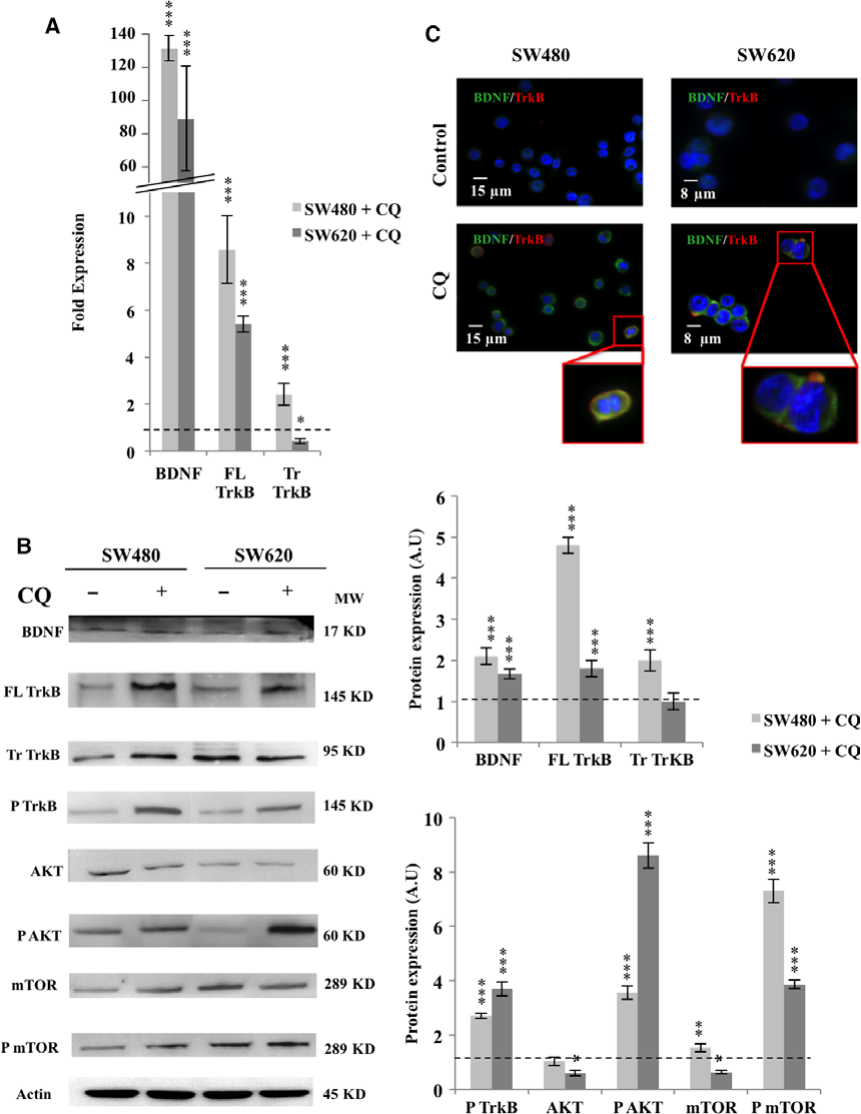
Pharmacologic inhibition of autophagy with CQ induced TrkB/BDNF pathway activation in CRC cell lines. SW480 and SW620 were treated with 25 μM of CQ for 3 hrs, recovered, washed and total RNA and proteins were extracted (see Materials and methods). (**A**) BDNF, FL TrkB and Tr TrkB, transcripts expression was assessed by qPCR as described in Fig 1 A. (**B**) BDNF, TrkB, phospho‐TrkB, AKT, phospho‐AKT, mTOR and phospho‐mTOR proteins expression were assessed by Western blotting. The density of each band representative for protein expression was calculated with ImageJ software. Images show representative results of three experiments. Statistically results are explained in comparison with control cells, normalized at 1 (*P < 0.05; **P < 0.01; ***P < 0.001). (**C**) Staining of SW480 and SW620 was realized with anti‐BDNF antibody (green), anti‐TrkB antibody (red) and DAPI (blue). Images were obtained through confocal microscopy (magnification ×1000).

Autophagy was inhibited either pharmacologically with CQ or genetically with ATG5‐siRNA. The use of CQ increased significatively BDNF and full‐length TrkB transcripts regardless of the cell line (Fig. 2A), which was confirmed at the protein level for both proteins and cell lines (*P* < 0.001; Fig. 2B). Evaluation of secreted BDNF detected only slight enhancement in SW480 (data not shown), consistent with the ability of BDNF to bind to TrkB that we observed in both cell lines (Fig. 2C). The suggested downstream activation of the NTs signaling was confirmed by the increase of phosphorylated form of TrkB (×2.5 for SW480 and ×3.5 for SW620; *P* < 0.001), AKT (×4 for SW480 and ×9 for SW620; *P* < 0.001) and mTOR (×7 for SW480 and ×4 for SW620; *P* < 0.001; Fig. 2B).

**Figure 3:**
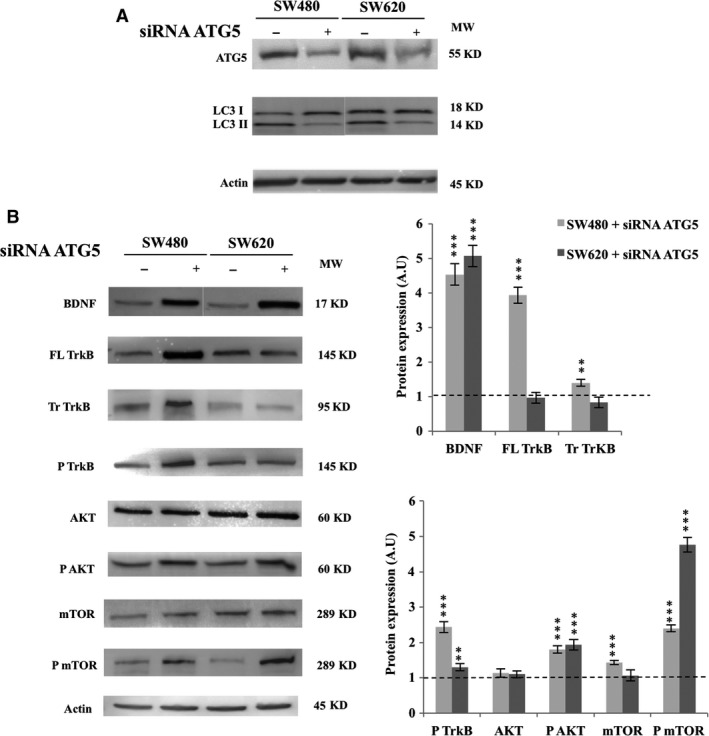
siRNA‐ATG5 inhibition of autophagy induced activation of the TrkB/BDNF pathway. SW480 and SW620 were transfected with siRNA ATG5, recovered, washed and total proteins were extracted (see Materials and methods). (**A**) To check silencing efficiency of siRNA, ATG5 and LC3 proteins expression were assessed by Western blotting. (**B**) BDNF, TrkB, phospho‐TrkB, AKT, phospho‐AKT, mTOR and phospho‐mTOR expression were assessed by Western blotting. The density of each band representative for protein expression was calculated with ImageJ software. Images show representative results of three experiments. Statistically results are explained in comparison with control cells, normalized at 1 (*P < 0.05; **P < 0.01; ***P < 0.001).

To avoid the potential side effects of CQ, autophagy was also inhibited using ATG5‐siRNA. The efficiency of siRNA was assessed by the decrease in ATG5 expression and LC3‐II/LC3‐I ratio (Fig. 3A: loss of almost 60% for SW480 and 50% for SW620; *P* < 0.001). As observed with CQ, the genetic autophagy downregulation increased expression of BDNF (×5 for SW480 and SW620; *P* < 0.001), phospho‐TrkB (×2.4 for SW480; *P* < 0.001 and ×1.2 for SW620; *P* < 0.01), and the downstream effectors phospho‐AKT (around ×2 for SW480 and SW620; *P* < 0.001) and phospho‐mTOR (×2.5 for SW480 and ×4.5 for SW620; *P* < 0.001; Fig. 3B). These results emphasize that NTs signaling was activated when autophagy was blocked, thereby ensuring survival. Moreover, this relay seemed to be more loaded in primary CRC‐derived cell lines (*i.e*. SW480) because of the special expression enhancement of FL TrkB and phopspho‐TrkB (*P* < 0.001).

### Targeting both the TrkB/BDNF and the autophagy pathways jeopardized CRC cell line survival

**Figure 4:**
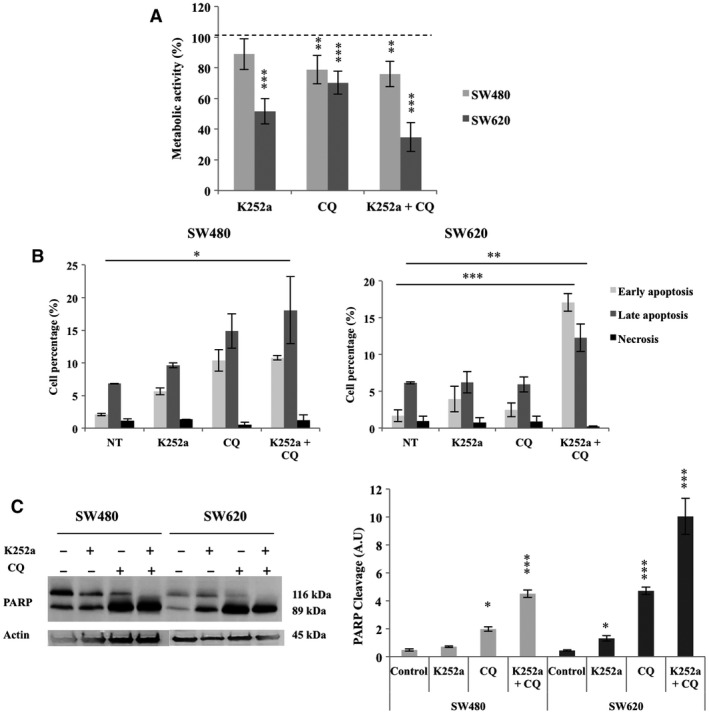
Metabolic activity, cell death analysis and PARP cleavage in CRC cell lines after K252a, CQ and K252a + CQ treatments. SW480 and SW620 were treated with K252a, CQ or both molecules for 72 hrs (see Materials and methods). (**A**) Metabolic activity was assessed through MTT testing. Histograms show the means ± S.E.M. of at least three independent experiments (*P < 0.05; **P < 0.01; ***P < 0.001). (**B**) Apoptosis and necrosis were determined by PI/Annexin‐V‐FITC method with flow cytometry. Histograms show the means ± S.E.M. of at least three independent experiments (*P < 0.05; **P < 0.01; ***P < 0.001). (**C**) PARP cleavage was assessed by Western blotting. The density of each band representative for protein expression was calculated with ImageJ software. Images show representative results of three experiments. Statistically results are explained in comparison with non treated cells, normalized at 1 (*P < 0.05; **P < 0.01; ***P < 0.001).

Because CRC cells used both the NTs and the autophagy to ensure their survival, dual inhibition was implemented to potentially sensitize cells to death. Although we failed to detect any change in cell fate after 3 hrs of treatment (data not shown), significant modifications were identified after 72 hrs (Fig. 4A). Even if the separate inhibition affected metabolic activity, it decreased more importantly with dual treatment (loss of 30% for SW480, *P* < 0.01, and loss of 65 % for SW620, *P* < 0.001). When evaluated the cell death pathway engaged by treated cells, we showed that the double pharmacological treatment significantly increased the apoptotic rate in both cell lines (×2.7 for SW480, *P* < 0.05 and ×3.7 for SW620, *P* < 0.001), whereas the necrotic death remained stable (Fig. 4B). These results were corroborated by the large enhancement of PARP cleavage (Fig. 4C) observed in both cell lines when treated with K252a + CQ (*P* < 0.01).

### Dual inhibition of TrkB/BDNF and autophagy induced a spectacular decrease in tumour growth

**Figure 5:**
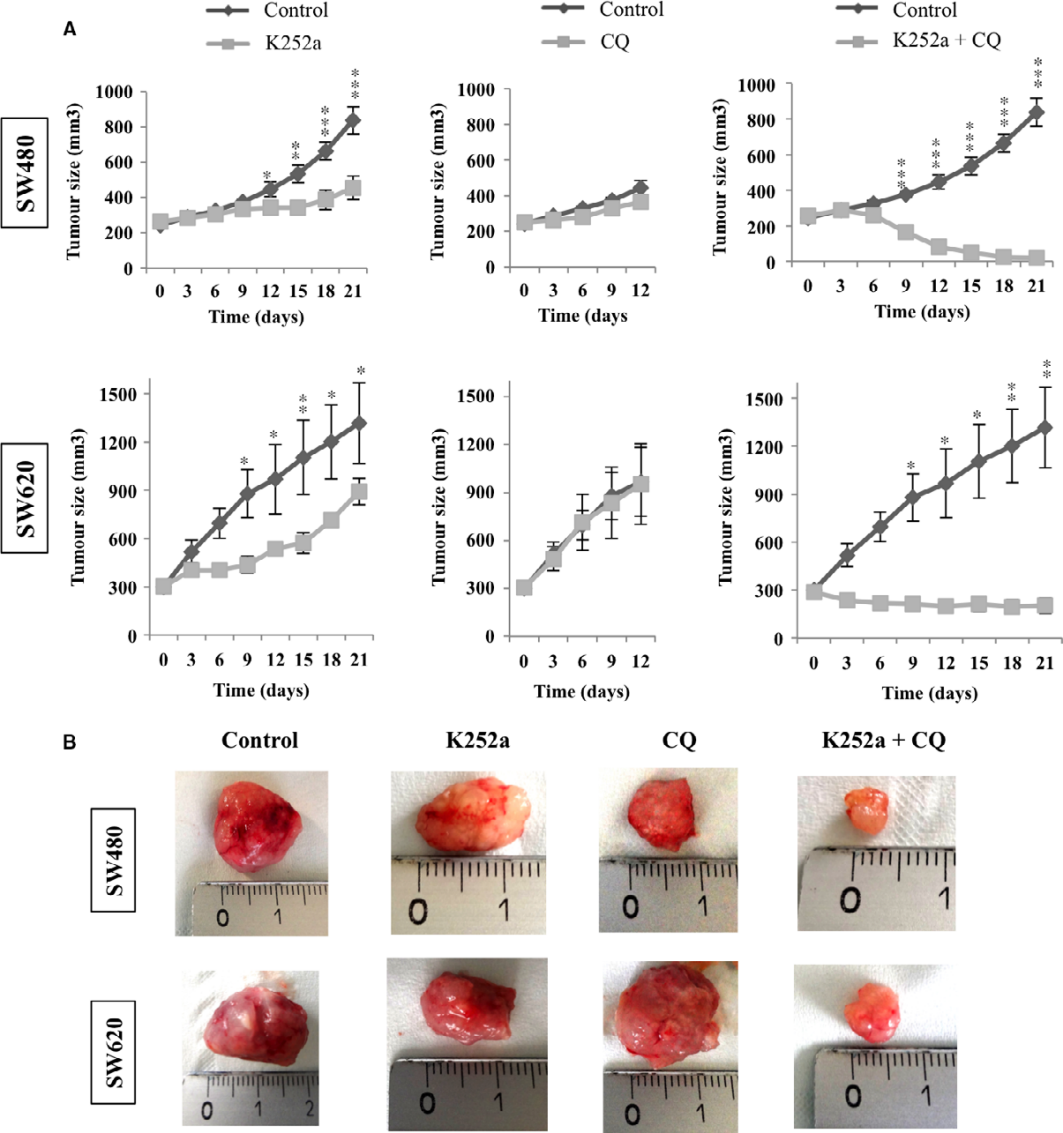
Effects of NT inhibitor (K252a), autophagy inhibitor (CQ) and dual treatment on tumoural growth. Nude mice were subcutaneously engrafted with SW480 or SW620 (1.10^6^ cells). When tumours reached a volume of 300 mm^3^, animals were divided into different groups and treated with K252a for 21 days or with CQ for 12 days or with both molecules (see Materials and methods). (**A**) Tumour growth was determined weekly. Results are expressed in mean tumour volumes (mm^3^) ± S.D. in comparison with the non treated group (*P < 0.05; **P < 0.01; ***P < 0.001). (**B**) Representative images of tumours.

Because the *in vitro* results indicated that dual inhibition was more effective than single inhibition, we checked efficiency of this dual treatment on *in vivo* tumours developed in nude mice. The K252a significantly decreased tumour volume compared with control xenografts in both cell lines after 21 days of treatment (loss of 50%, *P* < 0.001 for SW480 and loss of 45%, *P* < 0.05 for SW620; Fig. 5A and B). On another hand, the CQ did not induce a significant decrease in tumour volumes even after 12 days of treatment. At the opposite, the dual inhibition induced a significant reduction in tumour volume compared with control after 21 days of treatment (decrease of 95% for SW480, *P* < 0.001, and of 75% for SW620, *P* < 0.01). More surprisingly, 40% of the tumours in the case of SW480 and of 25% of the tumours in the case of SW620 totally disappeared. Finally, tumours from SW480 xenografts were more sensitive to dual treatment than those from SW620 xenografts.

**Figure 6:**
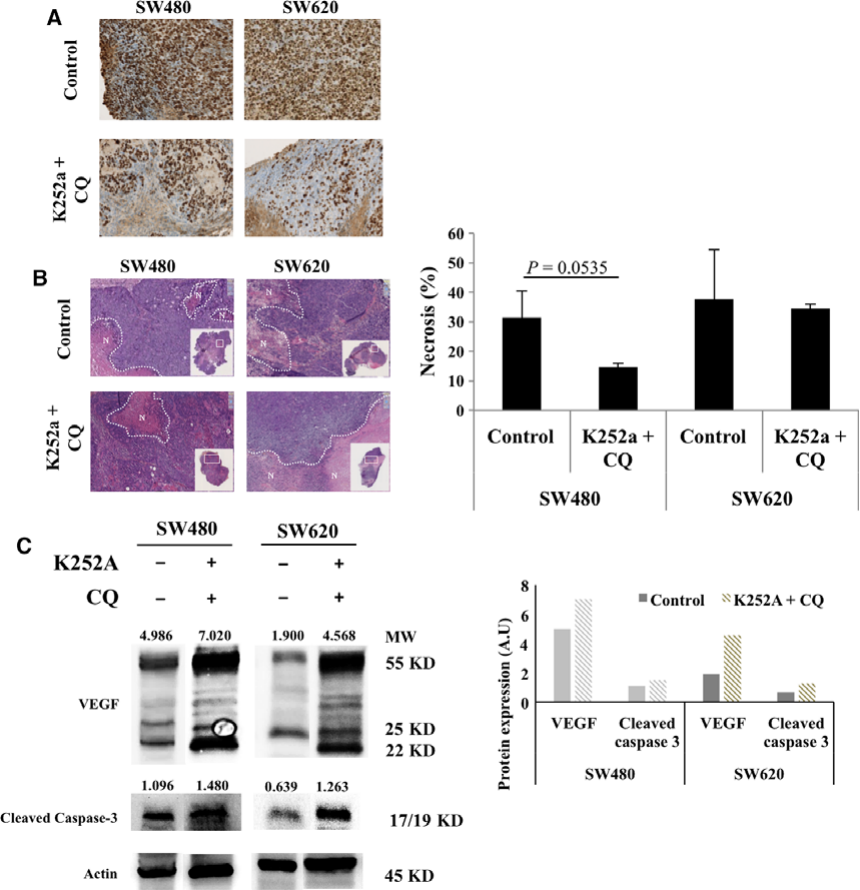
Effects of dual treatment (K252a + CQ) on cell proliferation, cell death and vasculogenesis in tumour tissue originating from mice SW480 and SW620 xenografts. After being engrafted subcutaneously by SW480 or SW620, mice were treated by K252a + CQ as described in Materials and methods section. Tumour sample were collected from five xenograft mice for both cell lines. (**A**) Proliferation was determined by Ki67 immunostaining. Magnification was ×100. (**B**) Necrosis was quantified using HES staining. Aeras of necrosis are delimited by N. Magnification was ×50. Results are representative of the ratio between necrosis area and total tumour area obtained with the Nanozoomer Digital Pathology 2.ORS (Hamamatsu) version 2.5.88 software. (**C**) Tumour sample were lysed and total protein were extracted. VEGF and cleaved caspase 3 were assessed by Western blotting. The density of each band was calculated with ImageJ software. Images show representative results performed for each condition, for both xenografts cell lines.

To delineate the mechanisms of tumour growth inhibition, we evaluated the relative rate of apoptosis and necrosis, as well as proliferation and angiogenesis by the mean of cleaved caspase 3 expression, HES and Ki67 staining and VEGF expression, respectively (Fig. 6). In both cell line engrafted tumours, we observed a proliferation decrease, as indicated by Ki67 expression reduction, when animals were double treated by K252a + CQ compared to controls (Fig. 6A). As HES staining revealed necrotic areas (Fig. 6B), we quantified the relative extent of the mechanism (Fig. 6B). Whereas the rate remained stable in SW620 engrafted tumours, it fell in double treated SW480 engrafted tumours, being borderline with significant threshold (*P* = 0.0535). Such observation could be explained by the great sensitivity of this cell line engrafted tumour, where we even observed a disappearance of some tumours. In the remaining tissue, necrotic areas could have been partially eliminated by host reaction. Furthermore, evaluation of apoptosis by cleaved caspase 3 expression showed a great increase in both cell lines engrafted tumours double treated compared to controls. Such results suggest that this cell death pathway was induced when autophagy and NT signaling were inhibited (Fig. 6C), corroborating *in vitro* data. Moreover, enhancement of VEGF expression with double treatment underlined the neo‐vascularization frequently occuring in CRC cancer and was compatible with a greater treatment accessibility (Fig. 6C).

### Relevance of neurotrophins and autophagy as potential targets in patients with CRC

**Figure 7:**
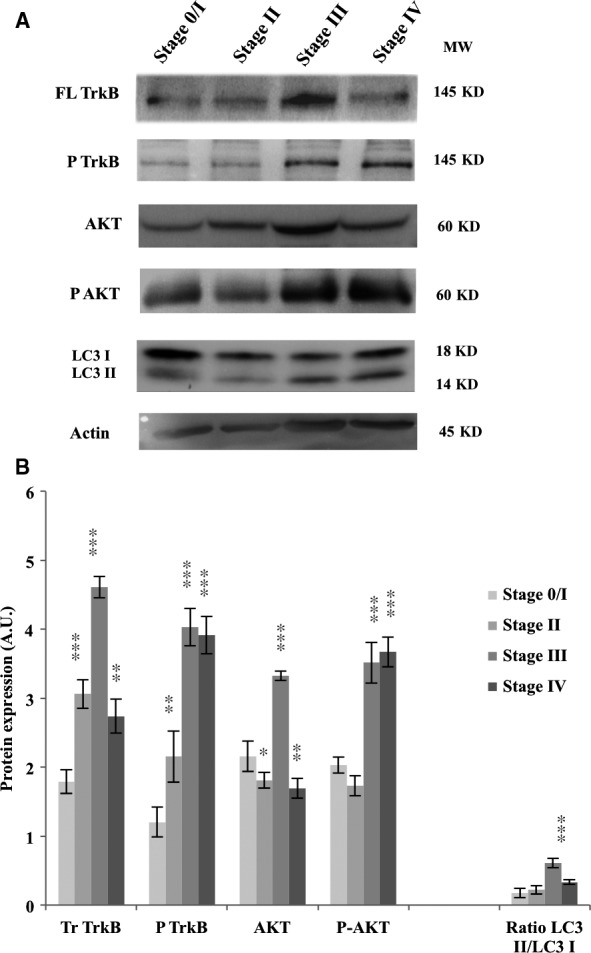
Neurotrophin and autophagy pathways are active in patients with CRC. Tumour samples from each stage were collected from at least three patients with CRC, lysed and total proteins were extracted. FL TrkB, phospho‐TrkB, AKT, phospho‐AKT and LC3 protein expression was assessed by Western blotting. The density of each band was calculated with ImageJ software. Images show representative results of at least three experiments. Significant differences were obtained by comparison with Stage 0/I, considered as a pre‐cancerous stage (*P < 0.05; **P < 0.01; ***P < 0.001).

To extend these results to patients with CRC, NTs and autophagy markers expression was evaluated in tissue samples obtained from different stage tumours (three patients per stage). An increase in phospho‐TrkB and phospho‐AKT was observed, highlighting the activation of these pathways (Fig. 7). Moreover, the phosphorylation rate seemed dependent on the stage of the CRC, with an increase observed for advanced stages, especially in stage III and stage IV (*P* < 0.001) compared to stage 0/I. In addition, the LC3II/LC3I ratio also varied depending on stage, exhibiting increases in advanced stages (Fig. 7). These data emphasize the specific high level of TrkB expression and activation, as well as the occurrence of autophagy in tumoural cells and corroborate the potential role of these signaling pathways in CRC aggressiveness.

## Discussion

**Figure 8:**
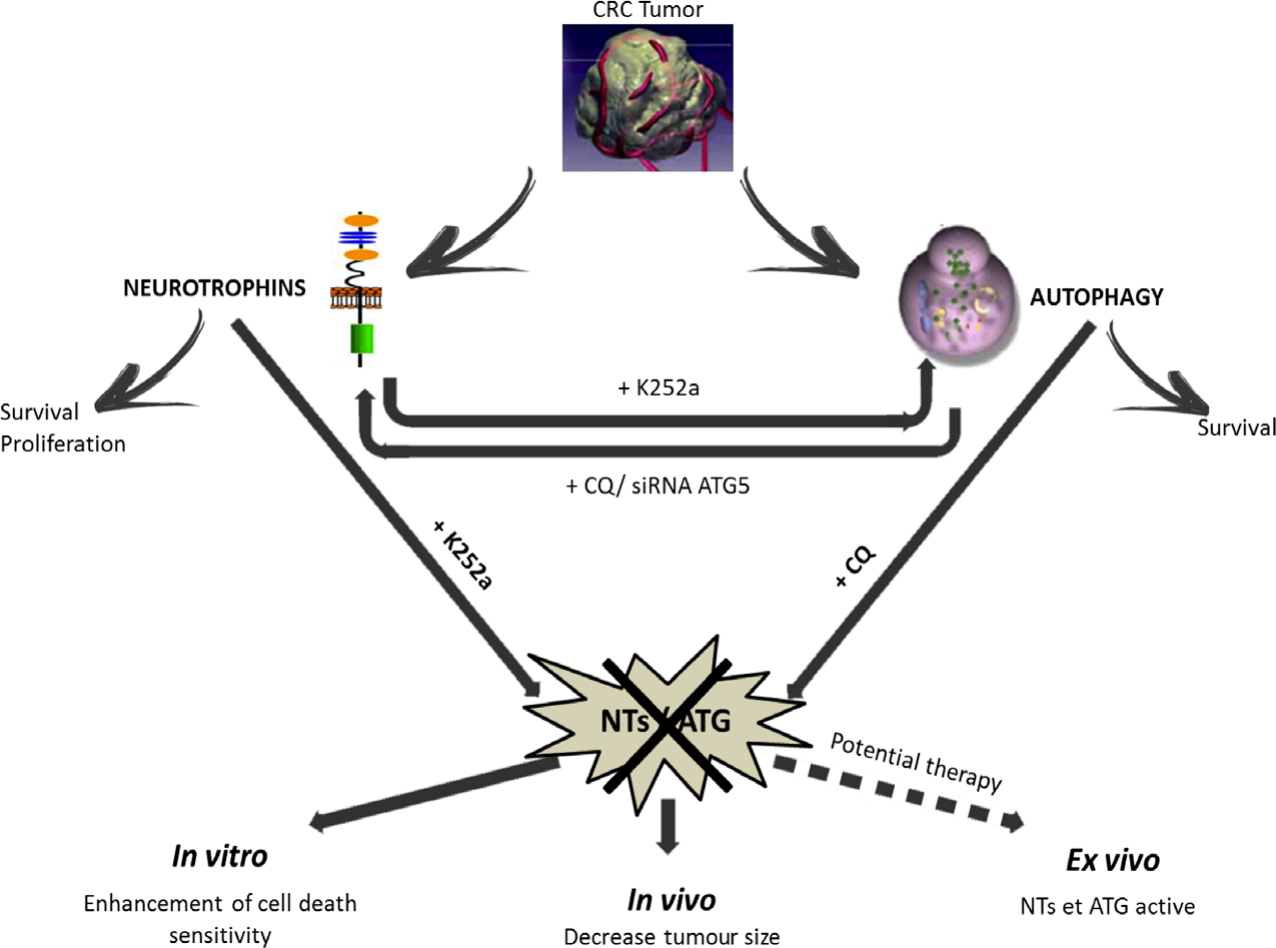
Take home message. Whereas single NT or autophagy inhibition failed to sensitive CRC cells to death and induce activation of the reciprocal pathway, the dual inhibition prompted *in vitro* cell death, *in vivo* reduction of tumour growth, thus representing a promising new therapeutic approach.

Because CRC stays a world problem, new therapeutic targets are largely needed. This study focuses on inhibition of NTs (TrkB/BDNF) and/or autophagy, which are two survival‐signaling pathways and shows the efficiency of the twin treatment, which largely contribute to reduce tumour growth (Fig. 8).

Overexpression of TrkB and BDNF constitute poor prognostic factors of tumour aggressiveness in various cancers 7, 10, 13, 36, 37. This axis is also associated with the proliferation, migration and invasion in the case of peritoneal carcinomatosis 38 and constitutes an autocrine and paracrine loop survival in stress response, as we and others previously demonstrated in CRC 7. In the present work, we used the pan‐Trk inhibitor (K252a), in the primary CRC‐derived cell line (SW480) and in the lymph‐node‐derived cell line (SW620). As both cell lines used in this work only expressed TrkB, this receptor signaling was the only one targeted. We observed the transcriptional and protein overexpression of TrkB and BDNF, which, even when binding together, failed to activate the AKT/mTOR pathway. This enhancement of both NT and receptor probably took place to try to restore this pathway. These results are consistent with previous reports, suggesting that K252a, although unable to block BDNF and TrkB interaction, is able to block downstream signaling 39. In another tumoural model—Ewing sarcoma—, this inhibitor was responsible of a proliferation and survival loss of tumoural cells by targeting both TrkA and TrkB receptors, 19. Even if the metastatic cell line SW620 showed comparable survival decrease, the reduced signaling in SW480 cells failed to induce a significant cytotoxicity, suggesting the involvement of a different cell survival process.

Among them, autophagy implication in CRC survival and in therapy resistance has already been shown 27, 28, 40, 41. Here, we showed that inhibition of NTs with K252a resulted in activation of autophagy to ensure cell survival. A similar protective effect of autophagy has also been reported when the EGFR pathway is inhibited with erlotinib in non‐small‐cell lung cancer 42 or with anti‐EGFR in CRC 43. Moreover, we described a similar compensatory effect of autophagy in response to hypoxia when TrkC signaling was reduced in glioblastoma (Jawhari *et al*., 2016, manuscript in revision). These results underscore the interplay between growth factors signaling (NTs) and autophagy.

Subsequently, we used either CQ, a classical autophagic flux‐blocking molecule 41, or siRNA ATG5 (to counteract the well‐known toxicity of CQ 44) to test the reciprocal effect of autophagy inhibition on TrkB/BDNF signaling. Both approaches increased NTs transcripts and proteins expression as well as subsequent AKT/mTOR signaling, demonstrating the activation of this pathway. Therefore, autophagy inactivation induced the activation of the TrkB/BDNF survival pathway.

The dual inhibition of NTs and autophagy potentiated the effects of the single molecules *in vitro*, especially to induce apoptosis but also, in a spectacular way, *in vivo* on tumour growth. When analysing the mechanisms of inhibition of tumour growth, we observed that size reduction was mainly du to proliferation impairment and apoptosis potentialization in both cell line engrafted tumours. However, as shown with HES staining, necrosis also took place and we cannot absolutely rule out involvement of this pathway in tumour reduction. Similar positive effects of dual targeting efficiency have been described *in vitro* and *in vivo* 43, 44, 45. For example, ongoing clinical trials already began in CRC, with the FOLFOX/bevacizumab/hydroxychloroquine study (clinical trial NCT01206530). Recently, the use of CQ was considered as an adjunct to chemotherapy 46 and inhibition of autophagy is one last option to overcome the RTK resistance 47, reinforcing the use of dual inhibition to fight cancer.

Our study also revealed a difference between SW480 and SW620, already described in terms of morphology, BrdU labelling index and apoptosis susceptibility 48. Especially we observed a higher level of basal TrkB and BDNF expression in primary tumour cells—SW480—than in metastatic cells—SW620—, that we and other previously reported 7, 49. Furthermore, SW480 presents a more important autophagy basal rate than their metastatic counterpart. We hypothesized that the primary tumour cell line emphasizes both survival pathway in order to resist to a lower nutritional supply, due to defective vascularization frequently observed in primary tumour from which they come from 50. In addition, relationship between TrkB expression and anoïkis evasion, which supports distant metastasis, has already been published in CRC 49, 51. This link could explain the behaviour difference in TrkB expression between the two cells lines, as SW480 metastasis gave raise to SW620. This enhanced activation of both autophagy and NT pathways is in accordance with SW480 higher sensitivity to twin inhibition especially in engrafted tumours. So, targeting both autophagy and NTs in CRC primary tumours will be more effective, as observed *in vivo*. Moreover, an increased resistance of SW620 against treatments also suggests that other signaling pathways might be activated, in accordance with their capacity to adapt their behaviour to different environment (*i.e*. lymph node area).

Finally, the presence of phospho‐TrkB and LC3II proteins was confirmed and increased with the stages of CRC in patient tissues. Because an excessive autophagic response was linked to metastasis and poor prognosis 52 and an increased TrkB expression enhanced the malignant potential in terms of proliferation, migration, invasion and anoikis inhibition in CRC cells 49, these two pathways constitute potential therapeutic targets, what our present study demonstrated.

## Conflict of interest

The authors declare that they have no conflicts of interest with the contents of this article.
